# Deep Cross-Corpus Speech Emotion Recognition: Recent Advances and Perspectives

**DOI:** 10.3389/fnbot.2021.784514

**Published:** 2021-11-29

**Authors:** Shiqing Zhang, Ruixin Liu, Xin Tao, Xiaoming Zhao

**Affiliations:** ^1^Institute of Intelligence Information Processing, Taizhou University, Zhejiang, China; ^2^School of Sugon Big Data Science, Zhejiang University of Science and Technology, Zhejiang, China

**Keywords:** speech emotion recognition, cross-corpus, deep learning, feature learning, survey

## Abstract

Automatic speech emotion recognition (SER) is a challenging component of human-computer interaction (HCI). Existing literatures mainly focus on evaluating the SER performance by means of training and testing on a single corpus with a single language setting. However, in many practical applications, there are great differences between the training corpus and testing corpus. Due to the diversity of different speech emotional corpus or languages, most previous SER methods do not perform well when applied in real-world cross-corpus or cross-language scenarios. Inspired by the powerful feature learning ability of recently-emerged deep learning techniques, various advanced deep learning models have increasingly been adopted for cross-corpus SER. This paper aims to provide an up-to-date and comprehensive survey of cross-corpus SER, especially for various deep learning techniques associated with supervised, unsupervised and semi-supervised learning in this area. In addition, this paper also highlights different challenges and opportunities on cross-corpus SER tasks, and points out its future trends.

## Introduction

Emotion recognition is an important direction in psychology, biology, and computer science, and has recently received extensive attention from the engineering research field. One of the starting points for emotion recognition is to assist in designing more humane human-computer interaction (HCI) methods, since emotion plays a key role in the fields of HCI, artificial intelligence (Cowie et al., 2001; Ramakrishnan and El Emary, 2013; Feng and Chaspari, 2020).

Traditional HCI is mainly carried out through keyboard, mouse, screen, etc. It only pursues convenience and accuracy, and cannot understand and adapt to people's emotions or mood. And if the computer lacks the ability to understand and express emotions, it is difficult to expect the computer to have the same intelligence as human beings. Moreover, it is also difficult to expect HCI to be truly harmonious and natural. Since the communications and exchanges between humans are natural and emotional, people naturally expect computers to have emotional capabilities in the procedure of HCI. The purpose of affective computing (Picard, 2010) is to endow computers the ability to observe, understand, and generate various emotional features similar to humans, and ultimately enable computers to interact naturally, cordially, and vividly like humans.

Emotion recognition is one of the most basic and important research subjects in the field of affective computing. Speech signals convey human emotional information most naturally. At present, speech emotion recognition (SER), which aims to classify human emotions from affective speech signals, has become a hot research topic in the fields of signal processing, pattern recognition, artificial intelligence, HCI, etc. Studying on SER has been going on for more than two decades (Schuller, 2018) and it has been applied to HCI (Cowie et al., 2001; Fragopanagos and Taylor, 2005), affective robots (Samani and Saadatian, 2012; Zhang et al., 2013), call-centers (Morrison et al., 2007), e-learning system (Li et al., 2007), computer games (Yildirim et al., 2011), depression severity classification (Harati et al., 2018), detection of autism spectrum disorder (ASD) (Lin et al., 2020), and so on.

During the past two decades, tremendous efforts have been made to focus on SER. Several survey related to SER can be found in El Ayadi et al. (2011), Anagnostopoulos et al. (2015), and Akçay and Oguz (2020). Note that the majority of existing SER systems are trained and evaluated on a single corpus and a single language setting. However, in many practical applications, there are great differences between training corpus and testing corpus. For example, the training and testing corpora come from two (or more) different languages, cultures, distribution modes, data scales, and so on. These differences across corpora result in significant idiosyncratic variations impeding the generalization of current SER techniques, thereby yielding an active research subject called cross-corpus SER in the field of SER.

Generally, in a basic cross-corpus SER system there are two crucial steps: emotion classifier and domain-invariant feature extraction. In the following, we will introduce these two steps of cross-corpus SER in brief.

As for emotion classifier, various traditional machine learning methods can be utilized for cross-corpus SER. The representative emotion classification methods contain linear discriminant classifier (LDC) (Banse and Scherer, 1996; Dellaert et al., 1996), K-Nearest Neighbor (Dellaert et al., 1996), artificial neural network (ANN) (Nicholson et al., 2000), support vector machines (SVM) (Kwon et al., 2003), hidden Markov models (HMM) (Nwe et al., 2003), Gaussian mixture models (GMM) (Ververidis and Kotropoulos, 2005), sparse representation classification (SRC) (Zhao and Zhang, 2015) and so on. Nevertheless, each classifier has its own advantages and disadvantages. The classifier combination method integrating the advantages of multiple classifiers (Morrison et al., 2007; Albornoz et al., 2011) began to draw researchers' attention.

Domain-invariant feature extraction, which aims to learn generalized feature representations of affective speech that are invariant across corpora, is another critical step in a cross-corpus SER system. So far, a variety of domain-invariant feature extraction methods have been explored for cross-corpus SER. According to the fact that the used data label information is whether included or not, existing domain-invariant feature extraction techniques for cross-corpus SER can be divided into three categories: supervised learning, semi-supervised learning, and unsupervised learning. Supervised learning is defined by its use of labeled sample data. In terms of labeled inputs and outputs, the used algorithm could measure its performance over time. In contrast, unsupervised learning aims to discover the inherent structure of unlabeled sample data without the demand for human intervention. Semi-supervised learning characterizes a type of the learning algorithms which try to learn from unlabeled and labeled sample data, generally supposing that the samples come from the same or similar distribution.

In the early cross-corpus SER literatures, to alleviate the problem of corpus-specific discrepancy for generalization, a variety of supervised, unsupervised, and semi-supervised techniques have been already developed on the basis of several typical hand-crafted low-level descriptors (LLDs), such as prosodic features, voice quality features and spectral features (Luengo et al., 2010; Zhang and Zhao, 2013), the INTERSPEECH-2009 emotion challenge (384 parameters) (Schuller et al., 2009b), the INTERSPEECH-2010 paralinguistic challenge (1,582 parameters) (Schuller et al., 2010a), the INTERSPEECH-2013 computational paralingusitics challengE (ComParE) set (6,373 parameters) (Schuller et al., 2013), the Geneva minimalistic acoustic parameter set (GeMAPS) (88 parameters) (Eyben et al., 2016), and so on. In particular, after extracting hand-crafted LLDs, for simply eliminating differences of cross-corpus acoustic features, corpus-based normalization in a supervised (Schuller et al., 2010b) or unsupervised manner (Zhang et al., 2011) was presented. In addition, several more sophisticated methods were also developed to learn common feature representations from the extracted hand-crafted LLDs, by means of supervised-based (Song et al., 2016b) or semi-supervised based matrix factorization (Luo and Han, 2019), supervised-based (Mao et al., 2017), or unsupervised-based domain adaption (Deng et al., 2017), etc. In recent years, the current state-of-art technique is to employ an adversarial learning scheme in an unsupervised (Abdelwahab and Busso, 2018) or semi-supervised (Latif et al., 2020) manner for learning a domain-invariant acoustic feature representation on cross corpus SER tasks.

Although the above-mentioned hand-crafted acoustic features associated with supervised, unsupervised, and semi-supervised learning approaches can produce good domain-invariant features for cross-corpus SER, they are still low-level and not highly discriminative. It is thus desirous to obtain high-level domain-invariant feature representations for cross-corpus SER.

To achieve high-level domain-invariant feature representations for cross-corpus SER, the recently-emerged deep learning (LeCun et al., 2015) methods may present a possible solution. The representative deep leaning techniques contain deep belief networks (DBNs) (Hinton and Salakhutdinov, 2006), convolutional neural networks (CNNs) (Krizhevsky et al., 2012), recurrent neural networks (RNNs) (Elman, 1990) and its variant called long short-term memory (LSTM) (Hochreiter and Schmidhuber, 1997), autoendcoders (AEs) (Ballard, 1987; Schmidhuber, 2015) and so on. So far, deep learning methods have shown good performance on object detection and classification (Wu et al., 2020), natural language processing (Otter et al., 2020), speech signal processing (Purwins et al., 2019), multimodal emotion recognition (Zhou et al., 2021), and so on, due to its strong feature learning ability.

Inspired by the lack of summarizing recent advances in various deep learning techniques for cross-corpus SER, this paper aims to present an up-to-date and comprehensive survey of cross-corpus SER, especially for various deep learning techniques associated with supervised, unsupervised and semi-supervised learning in this area. In addition, this paper highlights different challenges and opportunities on cross-corpus SER tasks, and point out its future trends. To the best of our knowledge, we are the first attempt to provide such a review for deep cross-corpus SER.

The organization of this paper is as follows. A review of speech emotion databases is presented at first. Then, we simply review supervised, unsupervised, and semi-supervised learning in details. Next, we review traditional methods for cross-corpus SER. We show recent advances of the applications of deep learning techniques incorporated with supervised, unsupervised and semi-supervised learning for cross-corpus SER. Next, we give a summary of open challenge and future directions. Finally, concluding remarks are provided.

## Speech Emotion Databases

For cross-corpus SER, a variety of speech emotion databases have been developed. Table 1 presents a brief summary of existing speech emotion databases. In this section, we describe briefly these existing speech emotion databases, as described below.

**Table 1:** A brief summary of speech emotion databases.

**Corpus/References**	**Language**	**Year**	**Categories**	**Size**	**Speakers**	**Recordings**	**Modalities**
DES/ (Engberg et al., 1997)	Danish	1997	Neutral, surprise, anger, happiness, sadness	5,200	4 (2f)	Acted	Audio
SUSAS/ (Hansen and Bou-Ghazale, 1997)	English	1997	Four states of speech under stress: neutral, angry, loud, Lombard	16,000	32 (13f)	Natural	Audio
SmartKom/ (Steininger et al., 2002)	German	2002	Neutral, joy, anger, helplessness, contemplation, surprise	3,823	70 (39f)	Natural	Audio
FAU-AIBO/ (Batliner et al., 2004)	German	2004	Anger, bored, emphatic, helpless, joyful, motherese, neutral	4,525	51 (30f)	Natural	Audio
EMO-DB/ (Burkhardt et al., 2005)	German	2005	Anger, boredom, disgust, fear, happiness, sadness, neutral	535	10 (5f)	Acted	Audio
eNTERFACE05/ (Martin et al., 2006)	English	2006	Anger, disgust, fear, happiness, sadness, surprise	1,277	42 (8f)	Elicited	Audiovisual
MASC/ (Wu et al., 2006)	Mandarin	2006	Neutral, anger, pride, panic, sadness	25,636	68 (23f)	acted	Audio
SAL/ (Douglas-Cowie et al., 2007)	English	2007	Anger, sadness, happiness, fear, neutral	1,692	4 (2f)	Natural	Audiovisual
ABC/ (Schuller et al., 2007)	German	2007	Aggressive, cheer, intoxicated, nervous, neutral, tire	431	8 (4f)	Elicited	audiovisual
CASIA/ (Zhang and Jia, 2008)	Mandarin	2008	Surprise, happiness, sadness, anger, fear, neutral	9,600	4 (2f)	Acted	Audio
VAM/ (Grimm et al., 2008)	German	2008	Dimension emotions (valence, arousal, dominance)	946	47 (32f)	Natural	audiovisual
IEMOCAP/ (Busso et al., 2008)	English	2008	Happiness, anger, sadness, frustration, neutral	1,150	10 (5f)	Elicited	Audiovisual
AVIC/ (Schuller et al., 2009a)	German	2009	Breathing, consent, garbage, hesitation, laughter	996	21 (10f)	Natural	Audiovisual
Polish/ (Staroniewicz and Majewski, 2009)	Polish	2009	Anger, sadness, happiness, fear, disgust, surprise, neutral	2,351	13 (7f)	Acted	audiovisual
IITKGPSEHSC/ (Koolagudi et al., 2011)	Hindi	2011	Happy, sad, angry, sarcastic, fear, neutral, disgust, surprise	1,200	10 (5f)	Acted	Audio
EMOVO/ (Costantini et al., 2014)	Italian	2014	disgust, fear, anger, joy, surprise, sadness	588	6 (3f)	Acted	Audiovisual
SAVEE/ (Jackson and Haq, 2014)	English	2014	Anger, sadness, fear, disgust neutral, joy, surprise	480	4 (-)	Acted	Audiovisual
AFEW/ (Dhall et al., 2015)	English	2015	Anger, disgust, fear, joy, neutral, sadness, surprise	1,645	330 (-)	Natural	Audiovisual
BAUM-1/ (Zhalehpour et al., 2016)	Turkish	2016	Happiness, anger, sadness, disgust, fear, surprise, boredom	1,222	31 (13f)	Natural	Audiovisual
MSP-IMPROV/ (Busso et al., 2017)	English	2017	Happiness, anger, sadness, neutral	8,438	12 (6f)	acted	Audiovisual
CHEAVD/ (Li et al., 2017)	Mandarin	2017	Anger, anxious, disgust, happiness, neutral, sadness, surprise, worried	2,852	238 (125f)	Natural	Audiovisual
NNIME/ (Chou et al., 2017)	Mandarin	2017	Discrete emotions (angry, happy, sad, neutral, frustration, surprise) and dimension emotions (valence, arousal, dominance)	102	44 (22f)	Acted	Multimodal
URDU/ (Latif et al., 2018a)	Urdu	2018	angry, sad, neutral, happy	400	38 (11f)	Natural	Audiovisual
RAVDESS/ (Livingstone and Russo, 2018)	English	2018	Calm, happy, sad, angry, fearful, surprise, disgust	7,356	24(12f)	Acted	Audiovisual
MSP-PODCAST/ (Lotfian and Busso, 2019)	English	2019	Discrete emotions (anger, sadness, happiness, surprise, fear, disgust, contempt and neutral) and dimension emotions (valence, arousal, dominance)	2,317	197 (87f)	Natural	Audio

### DES

The Danish Emotional Speech (DES) (Engberg et al., 1997) dataset contains 5,200 audio utterances, simulated by four professional actors (2 females, 2 males). The simulated utterances consist of five emotional states: anger, happiness, neutral, sadness, and surprise. The audio recordings from each actor are composed of two isolated words, nine sentences and two passages of fluent speech materials. The whole audio utterances last about 30 min in duration. For a listening test, 20 listeners were employed.

### SUSAS

The Speech Under Simulated and Actual Stress (SUSAS) (Hansen and Bou-Ghazale, 1997) dataset is a speech under stress corpus including five kinds of stress and feelings. It contains a highly confused collection of 35 aircraft communication vocabulary words. The researchers invited 32 speakers (13 females, 19 males) to produce more than 16,000 utterances. Simulated speech under stress is composed of ten stress styles such as speaking style, single tracking task, and Lombard effect domain.

### SmartKom

The SmartKom (Steininger et al., 2002) dataset is a multimodal corpus consisting of Wizard-Of-Oz dialogues in German and English from 70 subjects (31 males and 39 females). This dataset includes several audio tracks and two video tracks (face, side of body). The main purpose of this dataset is to conduct empirical researches on human-computer interaction in a variety of tasks and technological settings. This dataset contains several sessions, each of which has a one-person recording of about 4.5 min. All the collected 3,823 utterances were annotated with seven emotional states: neutral, joy, anger, helplessness, contemplation, surprise.

### FAU-AIBO

The FAU-AIBO (Batliner et al., 2004) corpus was collected from the recordings of children interacting with the Aibo pet robot. This dataset consists of spontaneous German speech. The children were made to believe that Aibo was reacting to their orders, while the robot was effectively controlled by a human operator. This dataset were obtained from 51 children (21 males, 30 females) ranging from 10 to 13 years old. The audio was recorded by using a DAT recorder (16-bit, 16 kHz). The audio recording is automatically segmented into “tums” using a 1 s pause. Five annotators were asked to listen to the tums in order and label each word individually as neutral (default) or the other ten categories. For annotation, the majority voting (MV) was employed. Finally, the utterance number for MV is 4,525, and contains 10 affective states: happy, surprise, stressed, helplessness, sensitivity, irritation, anger, mother, boredom, and condemnation.

### EMO-DB

The Berlin emotional speech database (EMO-DB) (Burkhardt et al., 2005), covers seven emotional states: anger, boredom, disgust, fear, happiness, neutral, and sadness. Verbal contents come from 10 German (5 males and 5 females) pre-defined neutral utterances. Ten professional actors were invited to speak each utterance in all seven emotional states. EMO-DB consists of approximately 535 sentences from seven emotions. The audio files were recorded with a sampling rate of 16 kHz and a 16-bit resolution and mono channel. The duration for all audio files are average 3 s.

### MASC

The Mandarin affective speech corpus (MASC) (Wu et al., 2006) consists of 68 native speakers (23 women, 45 man) and five affective states: neutral, anger, pride, panic and sadness. Each participant reads 5 phrases and 10 sentences for 3 times for every emotion, thereby yielding 25,636 utterances. These sentences involves in all the phonemes in Chinese language. The purpose of this corpus is to investigate the prosody and linguistic information of affective expressions in Chinese. Additionally, prosody feature analysis and speaker identification baseline experiments were also carried out.

### eNTERFACE05

The eNTERFACE05 (Martin et al., 2006) corpus is an audio-visual video database which includes six elicited emotions: anger, disgust, fear, joy, sadness, and surprise. It is composed of 1,277 audio-visual video samples from 42 participants (8 females) with 14 different countries. Every participant was demanded to listen to six consecutive short tales, which were designed to invoke a particular feeling. Two experts were asked to determine whether the induced emotional response clearly characterizes the expected emotion.

### SAL

The Belfast Sensitive Artificial Listener (SAL) (Douglas-Cowie et al., 2007) corpus is a subset of the developed HUMAINE database. The used SAL subset (Wöllmer et al., 2008) includes 25 recording sessions from 4 speakers (2 men and 2 women). The average duration of each session is 20 min. These audio-visual recordings in this dataset were collected from natural man-machine sessions developed by a SAL interaction. Four annotators were employed to continually mark the real-time data based on the Feeltrace tool (Cowie et al., 2000). These 25 recording sessions were divided into turns in terms of energy-based voice activity detection, yielding a total of 1,692 turns.

### ABC

The Airplane Behavioral Corpus (ABC) (Schuller et al., 2007) is an audio-visual emotional database, which is designed for particular applications to public transportation. In order to elicit a certain emotion, a script was utilized to make the subject enter into the context of the guided storyline. The selected public transportation contains holiday flights with return flights related to serving of wrong food, tumultuous currents, falling asleep, talking to neighbors and so on. Eight gender-balanced participants between the ages of 25–48 years were invited to take part in the audio recording with the German language. After pre-segmentation by three experienced male annotators, a total of 11.5 h of video with 431 clips was collected. The mean duration of all 431 video clips is 8.4 s.

### VAM

The VAM (Vera-Am-Mittag) corpus (Grimm et al., 2008) contains audio-visual transcripts collected from the German television talk show, which was recorded in unscripted and spontaneous discussions. This dataset consists of 946 utterances collected from 47 guests (15 males and 32 females) of talk show. The discussion themes were related to private problems, including friendship crises, fatherhood, or happy events. To annotate speech data, the audio recordings were segmented into the utterance-level, making each utterance include at least one phrase. A certain number of human annotators were employed for labeling data (17 annotators for half of all the data, 6 annotators for the others).

### CASIA

The CASIA corpus (Zhang and Jia, 2008), developed by the institute of Automation, Chinese Academy of Science, consists of 9,600 audio files in total. This dataset contains six emotional states: happiness, sadness, anger, surprise, fear, and neutral. Four professional actors (two males and two females) were asked to simulate these emotions.

### IEMOCAP

The Interactive Emotive Binary Motion Capture Database (IEMOCAP) (Busso et al., 2008) was developed by the team of speech analysis and interpretation laboratory (SAIL) from the University of Southern California (USC). This dataset contains five sessions lasting around 12 h, and 1,150 utterances in total. They were collected from 10 professional actors in dyadic sessions whose faces, heads, and hands were marked in scripted and natural verbal interaction scenarios. The actors performed chosen affective scripts and elicited five emotions (happiness, anger, sadness, frustration, and neutral states) under the designed imaginary settings.

### AVIC

The Audio-Visual interest corpus (AVIC) (Schuller et al., 2009a) is an audio-visual emotional dataset designed for commercial applications. In this commercial scenario, the product demonstrator leads one of 21 subjects (10 women) by means of an English business presentation. The level of interest was annotated for each sub-speaker. In addition, the conversation content and non-verbal vocalization were also annotated in the AVIC collection. Finally, only 996 phrases with high inter-annotator agreement were obtained.

### Polish

The Polish (Staroniewicz and Majewski, 2009) corpus is a spontaneous emotional speech dataset with six affective states: anger, sadness, happiness, fear, disgust, surprise and neutral. This dataset was recorded by three groups of speakers: professional actors, amateur actors and amateurs. A total of 2,351 utterances were recorded in which 1,168 with female and 1,183 with male voice. The average duration of all utterances was about 1 s. Then, 202 listeners were invited to attend the listening tests, in which 33 of them were musically educated and 27 foreigners did not know the Polish language.

### IITKGP-SEHSC

The Indian Institute of Technology Kharagpur Simulated Emotional Hindi Speech Corpus (IITKGP-SEHSC) (Koolagudi et al., 2011) is an affective song and spoken corpus for the Hindi language. This dataset comprises of 10 participants (5 males, 5 females), each of which speaks 15 utterances in 10 sessions. It contains 1,200 audio files from 8 emotions: joy, sadness, anger, sarcasm, fear, neutral, disgust, surprise.

### EMOVO

The EMOVO (Costantini et al., 2014) corpus is the first affective dataset for the Italian language. This dataset was established by six professional actors who speak 14 sentences to simulate seven affective states: disgust, fear, anger, joy, surprise sadness, and neutral. These utterances were recorded with specialized facilities in the Ugo Bordoni laboratory. This corpus also presents a subjective verification test based on the emotion-classification of two sentences conducted by two different groups of 24 listeners.

### SAVEE

The Surrey audio-visual expression of emotion (SAVEE) (Jackson and Haq, 2014) corpus is a multimodal acted affective dataset with the British English language. It contains a total of 480 utterances with seven different emotions: neutral, happy, sad, angry, surprise, fear, and disgust. These utterances produced by four professional male actors. To keep the good quality of the affective acting, all the recordings in this dataset were verified by ten different evaluators under audio, visual and audio-visual condition. The scripts in these recordings were chosen from the conventional TIMIT corpus (Garofolo et al., 1993).

### AFEW

The Acted Facial Expressions in the Wild (AFEW) is a natural audio-visual affective video corpus which is provided for emotion recognition in the wild (EmotiW) challenge. There have been various recently-developed versions of AFEW datasets (Kossaifi et al., 2017). One of the popular AFEW datasets is AFEW5.0 (Dhall et al., 2015) collected from 330 speakers in 2015. AFEW5.0 consists of seven affective states: anger, disgust, fear, joy, neutral, sadness and surprise, evaluated by 3 annotators. AFEW5.0 contains 1,645 utterances in total and is split into three parts: the training set (723 samples), the validation set (383 samples), and the testing set (539 samples).

### BAUM-1

The BAUM-1 (Zhalehpour et al., 2016) audio-visual corpus is a spontaneous emotional dataset containing eight emotions (joy, anger, sadness, disgust, fear, surprise, boredom, and contempt), and four mental states (unsure, thinking, concentrating, and bothered). This dataset consists of 1,222 audio-visual samples from 31 Turkish participants (17 female, 14 males). The average duration of the whole samples is about 3 s. Five annotators were invited to label each sample by means of a majority voting.

### MSP-IMPROV

The MSP-IMPROV (Busso et al., 2017) acted database is an audio-visual affective dataset that records the English interaction of 12 actors (6 males, 6 females) in binary conversations. Each conversation is manually split into speech turns. It consists of 8,438 emotion sentences over 9 h from four emotions: happiness, anger, sadness, and neutral. At least 50,000 evaluators were recruited by using crowdsourcing to annotate these emotional contents. The audio recording rate was 48 kHz.

### CHEAVD

The CASIA Natural Emotion Audiovisual Data (CHEAVD) (Li et al., 2017) contains 2,852 natural emotional clips with 140 min extracted from 238 speakers (113 males, 125 females). This dataset is collected from 34 films, 2 television series, and 4 other television programs. This dataset is divided into three parts: the training set (1981), validation set (243) and testing set (628). The average duration of the whole samples is 3.3 s. It consists of eight emotional categories, such as angry, happy, sad, worried, anxious, surprise, disgust, and neutral. The sampling rate of audio files is 41 kHz.

### NNIME

The NTHU-NTUA Chinese Interactive Emotion Corpus (NNIME) (Chou et al., 2017) is a multimodal spontaneous emotional database, collected from 44 speakers (22 females, 22 males), involved in spontaneous dyadic spoken interactions. This dataset contains 102 dyadic interaction sessions with ~11 h of audio-video data. These participants come from the Department of Drama at National Taiwan University of Arts. Another 49 annotators were invited to implement a rich set of emotion annotations on discrete and dimensional annotation (valence, arousal, dominance). For discrete emotions, there are six categories: angry, happy, sad, neutral, frustration, surprise. The sample rate of audio recordings is 44.1 kHz.

### URDU

The URDU corpus (Latif et al., 2018a) is an unscripted and natural emotional spoken dataset with the first URDU language. It consists of 400 audio samples in four affective states (angry, happy, sad and neutral). In this dataset, the audio recordings were collected from the conversations of 38 participants (27 males and 11 females) on the Urdu television talk shows. Four different annotators were requested to make annotations for all the audio recordings based on the audio-visual condition.

### RAVDESS

The RAVDESS dataset (Livingstone and Russo, 2018) is a multimodal corpus of affective speech and songs. This dataset is gender-balanced and comprises 24 specialized actors (12 males, 12 females) who produce speech and song samples in a neutral North American pronunciation. For affective speech, it consists of calm, joy, sadness, anger, fear, surprise, and disgust. For affective songs, it consists of calm, joy, sadness, anger, fear, surprise, and disgust and fear. Every expression is generated at two levels of affective intensity with an additional neutral expression. The final collection of 7,356 recordings was individually rated for 10 times on these aspects of affective validity, intensity, and genuineness. For these ratings, 247 untrained research subjects from North America were employed.

### MSP-PODCAST

The MSP-PODCAST (Lotfian and Busso, 2019) natural corpus contains 2,317 utterances collected from 403 podcasts. These utterances come from 197 speakers' (110 males, 87 females) spontaneous English speech in the Creative Commons authorized recording downloaded from the audio sharing websites. These podcasts are evaluated by using crowdsourcing to be dimensional emotions (valence, arousal, dominance) and discrete emotions including anger, sadness, happiness, surprise, fear, disgust, contempt, and neutral. In total, 278 different workers are invited to evaluate these utterances. Audio recordings have a sampling rate of 8 kHz.

## Review of Supervised, Unsupervised, and Semi-Supervised Learning

In this section, we will simply review the concept and typical supervised, unsupervised, and semi-supervised learning techniques, as described below.

### Supervised Learning

Supervised learning usually requires a large number of labeled samples to carefully train the model for achieving better model generalization ability (Cunningham et al., 2008). At the same time, due to the problem of dimension disaster, when processing high-dimensional data, the number of labeled samples required to train a good supervised model will further show an exponential explosion trend. This makes it difficult for traditional supervised learning to be applied to some tasks that lack training samples. Nevertheless, supervised learning methods are usually simpler than unsupervised learning methods. Therefore, when training a supervised model, how to reduce the demand for labeled samples and improve the performance of model learning has become an important research problem (Alloghani et al., 2020).

Supervised learning can be further grouped into classification and regression. A classification problem is to deal with categorical outputs, whereas a regression problem is to process continuous outputs. The typical supervised learning methods contains ANN, SVM, HMM, GMM, random forest, Bayesian networks, decision tree, linear regression, logistic regression, and so on (Kotsiantis et al., 2007; Sen et al., 2020).

### Unsupervised Learning

Unlike supervised learning with labeled data, unsupervised learning aims to extract inherent feature representations from unlabeled sample data. Therefore, unsupervised learning mainly relies on previously learned knowledge to distinguish likely classes within unlabeled sample data. As a result, unsupervised learning is very appropriate for feature learning tasks (Alloghani et al., 2020).

In general, unsupervised learning methods can be divided into three categories (Usama et al., 2019): hierarchical learning, data clustering, and dimensionality reduction. Hierarchical learning aims to learn complicated feature representations from a hierarchy of multiple linear and non-linear activation operations. Autoencoders (AEs) (Ballard, 1987; Schmidhuber, 2015) are one of the earliest unsupervised hierarchical learning algorithms. Data clustering is a well-known unsupervised learning task that concentrates on seeking hidden patterns from input unlabeled sample data in the form of clusters. Data clustering methods can be grouped into three categories (Saxena et al., 2017): hierarchical clustering, Bayesian clustering, and partitional clustering. One of the widely-used data clustering approaches is k-means clustering (Likas et al., 2003) which belongs to partitional clustering. Dimensionality reduction (also called subspace learning) aims to seek the hidden pattern of the underlying data by means of extracting intrinsic low-dimensional structure. Dimensionality reduction can be categorized into two types: linear and non-linear methods (Van Der Maaten et al., 2009). Principal component analysis (PCA) (Wold et al., 1987) and non-negative matrix factorization (NMF) (Lee and Seung, 1999) are two popular linear dimensionality reduction methods.

### Semi-supervised Learning

In order to make full use of the advantages of unsupervised learning and supervised learning, semi-supervised learning aims to combine a small number of labeled data and a large number of unlabeled data for performing certain learning tasks. The main goal of semi-supervised learning is to harness unlabeled data for constructing better learning procedures. For example, for a classification problem, additional sample data without label information can be utilized to aid in the classification process for performance improvement.

Semi-supervised learning can be divided into two main types (van Engelen and Hoos, 2020): inductive and transductive methods. Inductive methods aim to construct a classification model that can be utilized to predict the label of previously unseen sample data. In this case, unlabelled data may be employed when training this classification model. The representative inductive methods (Ligthart et al., 2021) contain self-training, co-training, multi-view learning, generative models, and so on. Different from inductive methods, transductive methods do not need to build a classifier for the whole input space. The typical transductive methods are graph-based semi-supervised learning algorithms (Chong et al., 2020) in which they attempt to transfer the label information of a small set of labeled data to the remaining large unlabeled data with the aid of a graph. The popular graph-based semi-supervised learning algorithms include the graph Laplacian (Fergus et al., 2009), graph-based semi-supervised neural network models (Alam et al., 2018) like graph convolutional networks (Chen et al., 2020).

## Traditional Methods for Cross-Corpus SER

From the view of point of supervised, unsupervised, and semi-supervised learning, in this section we will introduce traditional methods for cross-corpus SER, as described below.

### Supervised Learning for Traditional Methods

On supervised cross-corpus SER tasks, researchers usually combine one or more databases as training sets and testify the performance on each labeled database as a testing set in a cross-validation scheme. In early supervised cross-corpus SER, the typical hand-crafted acoustic features and conventional classifiers were employed in a supervised learning manner. For instance, in Schuller et al. (2010b), they extracted 93 LLD features such as prosody, voice quality and articulatory features and performed speaker-corpus normalization so as to deal with the differences among corpora. Then, the linear SVM was used to conduct cross-corpus evaluation experiments. They adopted different combinations of training and testing sets on all used labeled databases for cross-corpus experiments. In Feraru et al. (2015), 1,941 LLD acoustic features like prosody, voice quality and spectral features were derived, then the linear SVM was employed for cross-corpus SER. A post-processing of the trained SVM models was performed by rule-based model inversion to cope with the difference among corpora. For cross-corpus experiments, they trained and tested each used labeled database against each. Based on the extracted INTERSPEECH-2010 Paralinguistic Challenge feature set with 1,582 LLDs, a new method of transfer non-negative matrix factorization (TNMF) (Song et al., 2016b), in which the non-negative matrix factorization (NMF) and the maximum mean discrepancy (MMD) algorithms were combined, was developed for cross-corpus SER. They also trained and tested each other for all used labeled database. They showed that the performance of the proposed TNMF was much better than the baseline method with the linear SVM. A domain adaptation based approach, named emotion-discriminative and domain-invariant feature learning method (EDFLM) (Mao et al., 2017), was presented for cross-corpus SER. Training and testing each other for all used labeled database was implemented. In this method, domain discrepancy was minimized, whereas emotion-discrimination was employed to produce emotion-discriminative and domain-invariant features, followed by the linear SVM for SER. They extracted the INTERSPEECH-2009 Emotion Challenge feature set as inputs of EDFLM. In Kaya and Karpov (2018), they provided a cascaded normalization method, integrating linear speaker level, non-linear value level and feature vector level normalization, and then employed an extreme learning machine (ELM) classifier for cross-corpus SER. Here, they extracted the ComParE feature set with 6,373 LLDs. They conducted cross-corpus experiments in two settings: single corpus training (one-vs.-one), and multiple corpus training via leave-one-corpus-out (LOCO) setting. A non-negative matrix factorization based transfer subspace learning method (NMFTSL) (Luo and Han, 2020), in which the knowledge of the source data could be transferred to the target data, was developed to seek a shared feature subspace for the source and target corpus on cross-corpus SER tasks. They extracted the INTERSPEECH-2010 Paralinguistic Challenge feature set and then adopted the linear SVM for cross-corpus SER. Based on all the used databases, they constructed 30 cross-corpus SER schemes by using multiple combinations for source and target corpus on cross-corpus SER task.

### Unsupervised Learning for Traditional Methods

For unsupervised cross-corpus SER tasks, researchers tried to investigate how agglomeration of unlabeled data. For instance, in Zhang et al. (2011) they extracted 39 functionals of 56 acoustic LLDs, yielding 6,552 features in total, and then employed the linear SVM to conduct a cross-corpus LOCO strategy for experiments. To evaluate the effectiveness of normalization techniques before data agglomeration, they investigated the performance of centering, normalization and standardization for per corpus normalization. Experiment results on multiple databases showed that adding unlabelled emotional samples to agglomerated multi-corpus training sets could improve SER recognition performance. To mitigate the different feature distributions between the source and target speech signals, a domain-adaptive subspace learning (DoSL) approach (Liu et al., 2018) was presented to learn a project matrix for yielding similar feature distributions. They utilized the INTERSPEECH-2009 feature set with 384 features and adopted the linear SVM for cross-corpus LOCO SER experiments. Likewise, to reduce the disparity of source and target feature distributions, a transfer subspace learning (TRaSL) (Liu et al., 2021) was also proposed for cross-corpus SER. The proposed TRaSL aimed to find a projection matrix which transformed the source and target speech signals into a common feature subspace. Finally, they adopted the INTERSPEECH-2009 feature set and the linear SVM for cross-corpus LOCO SER experiments.

### Semi-supervised Learning for Traditional Methods

For semi-supervised cross-corpus SER, some recent literatures have focused on the combination of unlabeled and labeled sample data for performance improvement. In particular, a new transfer learning technique, namely transfer semi-supervised linear discriminant analysis (TSDA) (Song et al., 2016a), was provided to produce corpus-invariant discriminative feature representations on cross-corpus SER tasks. They obtained the INTERSPEECH-2010 Paralinguistic Challenge feature set, and then performed cross-corpus SER with the linear SVM. They conducted cross-corpus experiments with a LOCO scheme, and showed that TSDA outperformed other methods. A semi-supervised adaptation regularized transfer non-negative matrix factorization (SATNMF) (Luo and Han, 2019) was presented to extract common features for cross-corpus SER. The proposed SATNMF method aimed to integrate the label information of training data with NMF, and found a latent low-rank feature space to minimize simultaneously the marginal and conditional distribution differences among several language datasets. They employed the ComParE feature set and the linear SVM for LOCO SER experiments.

In summary, Table 2 presents a summary of the above-mentioned supervised, unsupervised, and semi-supervised learning literatures for traditional methods on cross-corpus SER tasks.

**Table 2:** A brief summary of traditional cross-corpus SER literatures.

**References**	**Category**	**Input features**	**Methods for cross-corpus**	**Datasets**
Schuller et al. (2010b)	Supervised	93 LLDs	speaker-corpus normalization	DES/, EMO-DB, SUSAS, AVIC, SmartKom, eNTERFACE05
Feraru et al. (2015)	Supervised	1,941 LLDs	rule-based model inversion	EMO-DB, DES, eNTERFACE05
Song et al. (2016b)	Supervised	INTERSPEECH-2010	TNMF	FAU-AIBO, eNTERFACE05, EMO-DB
Mao et al. (2017)	Supervised	INTERSPEECH-2009	EDFLM	ABC, EMO-DB, FAU-AIBO
Kaya and Karpov (2018)	Supervised	ComParE	cascaded normalization	EMO-DB, DES, eNTERFACE05
Luo and Han (2020)	Supervised	INTERSPEECH-2010	NMFTSL	CASIA, SAVEE, EMO-DB, IEMOCAP, eNTERFACE05
Zhang et al. (2011)	Unsupervised	6,552 LLDs	corpus normalization	ABC, AVIC, DES, VAM, SAL, eNTERFACE05
Liu et al. (2018)	Unsupervised	INTERSPEECH-2009	DoSL	EMO-DB, eNTERFACE05
Liu et al. (2021)	Unsupervised	INTERSPEECH-2009	TRaSL	EMO-DB,eNTERFACE05, IEMOCAP
Song et al. (2016a)	Semi-supervised	INTERSPEECH-2010	TSDA	EMO-DB, eNTERFACE05
Luo and Han (2019)	Semi-supervised	ComParE	SATNMF	CASIA, EMO-DB, eNTERFACE05

## Deep Learning Methods for Cross-Corpus SER

From the view of point of supervised, unsupervised, and semi-supervised learning, in this section we will introduce deep learning methods for cross-corpus SER, as described below.

### Supervised Learning for Deep Learning Methods

For supervised cross-corpus SER with labeled databases, the typical CNN, LSTM, DBN, and its combinations in a hybrid way, associated with the transfer learning strategy, have been recently adopted. Specially, in Marczewski et al. (2017), to alleviate the different distributions of features and labels across domains, they proposed a deep learning network architecture composed of two uni-dimensional convolutional layers, one LSTM layer, and two FC layers for cross-corpus SER. The used CNN layers aimed to derive spatial features of varying abstract levels, whereas the LSTM layer was used to learn temporal information related to emotion evolution over time. In this case, they jointly exploited CNNs to extract domain-shared features and LSTMs to identify emotions with domain specific features. All the samples data from all databases were used for training and testing by using a 5-fold cross validation scheme. Experiments showed that they could learn transferable features to enable model adaptation from multiple source domains. In Latif et al. (2018b), considering the fact that DBNs have a strong generalization power, this study presented a transfer learning technique based on DBNs to improve the performance of SER in cross-language and cross-corpus scenarios. The used DBNs consisted of three RBM layers, in which the first two RBMs contained 1,000 hidden neurons, and the third RBM included 2,000 hidden neurons. The simple variant (eGeMAPS) of typical GeMAPS feature set, including 88 LLDs like pitch, energy, spectral, and so on, was employed as inputs of DBNs. For all used databases, a LOCO scheme was used for cross-corpus SER experiments. Experiment result demonstrated that DBNs provided better performance on cross-corpus SER tasks, compared with a SAE and the linear SVM. In Parry et al. (2019), after extracting 40 Mel filterbank coefficients, they presented a comparative analysis of the generalization capability of deep learning models like CNNs, LSTMs, and CNN-LSTM. The used CNNs were composed of one-dimension convolutional layer, and one max-pooling layer. The used LSTMs were two-layer bi-directional LSTMs. The used CNN-LSTM contained three CNNs and two LSTMs above-mentioned. This study indicated that the CNN and CNN-LSTM models gave very close performance, but better than LSTM. For cross-corpus experiments, all corpora were combined together, thereby producing 11 h 45 min for training, 1 h 30 min each for validation and testing. In Rehman et al. (2020), to develop a more adaptable SER in adversarial conditions, they presented a hybrid neural network framework for cross-corpus SER. The hybrid neural network consisted of two-layer LSTMs and a ramification layer. LSTMs aimed to learn temporal sequence data in the one-hot input matrices, yielded by the latter ramification layer. The ramification layer comprised of multiple embedding units and split the input MFCCs into subsequent one-hot output. They validated the performance of different methods by means of training deep models on two of the used databases and then testing it on the third database. Experiments showed the effectiveness of the proposed method on cross-corpus SER tasks.

### Unsupervised Learning for Deep Learning Methods

For unsupervised cross-corpus SER tasks by leveraging unlabeled data, the popular unsupervised autoendcoder (Ballard, 1987; Schmidhuber, 2015) and its variants have been widely employed. For instance, to address the discrepancy between training and testing data, an adaptive denoising autoencoder (A-DAE) based an unsupervised domain adaptation approach (Deng et al., 2014b) was developed for cross-corpus SER. In this method, the prior knowledge learned from a target set was utilized to regularize the training on a source set. When obtaining the INTERSPEECH-2009 Emotion Challenge feature set, A-DAE was employed to learn a common representation across training and test samples, followed by the linear SVM for cross-corpus SER. They conducted cross-corpus SER experiments by using a LOCO corpus scheme. In Deng et al. (2017), an end-to-end domain adaptation method, named universum autoencoder (U-AE), which retained feature representation ability to discover the intrinsic structure in input data, was presented for cross-corpus SER. The proposed U-AE aimed to enable the unsupervised learning autoencoder to have supervised learning ability, thereby improving the performance of cross-corpus LOCO SER. The standard INTERSPEECH-2009 Emotion Challenge feature set was employed as inputs of the proposed U-AE. This study indicated that the proposed U-AE outperformed other domain adaptation methods such as kernel mean matching (Gretton et al., 2009), and shared-hidden-layer autoencoders (Deng et al., 2014a). In Neumann and Vu (2019), they investigated how unsupervised representation learning on additional unlabeled data could be used to promote SER performance. More specially, they integrated feature representations learnt by using an unsupervised autoencoder into an attentive CNN-based emotion classifier so as to improve recognition performance on cross-corpus LOCO SER tasks. In detail, they firstly trained a recurrent sequence-to-sequence autoencoder on unlabeled data and then adopted it to produce feature representations for labeled target data. These produced feature representations were then incorporated as additional source information for emotion identification during the training process of the used attentive CNN.

In recent years, several advanced unsupervised learning methods such as adversarial learning (Goodfellow et al., 2014) and attentive learning have also been used for cross-corpus SER. Specially, in Abdelwahab and Busso (2018), a domain adversarial neural network (DANN), consisting of three parts: a feature representation layer, a task classification layer, and a domain classification layer, was employed to learn a common feature representation between training and testing data. DANN was trained by using labeled sample data in the source domain and unlabeled sample data in the target domain. The extracted acoustic features were the ComParE feature set as inputs of DANN. They conducted cross-corpus experiments by using single corpus training (one-vs.-one), and multiple corpus training via a LOCO scheme. This study demonstrated that adversarial training on the basis of unlabeled training data yielded an obvious performance improvement compared with training with the source data. In Ocquaye et al. (2021), a deep learning framework including three attentive asymmetric CNNs was presented to emotion identification for cross-lingual and cross-corpus speech signals in an unsupervised manner. They implemented cross-corpus SER experiments by using a LOCO corpus scheme. The proposed approach employed jointly supervised learning incorporated with softmax loss and center loss in order to learn high-level discriminative feature representations for target domain data with the aid of pseudo-labeled data. Evaluation results indicated that the proposed method outperformed a SAE and DBNs with three RBMs.

### Semi-supervised Learning for Deep Learning Methods

For semi-supervised cross-corpus SER by leveraging unlabeled and labeled data, adversarial learning (Goodfellow et al., 2014) was usually taken as a generative model for. For instance, in Chang and Scherer (2017), they explored a semi-supervised learning approach, called a multitask deep convolutional generative adversarial network (DCGAN), to improve cross-corpus performance. DCGAN was utilized to learn strong feature representation from the computed spectrograms on unlabeled data. For multitask learning, the proposed multitask model took emotional valence as a primary target and emotional activation as a secondary target. For evaluation, they combined unlabeled data from all used databases and testified the performance on one labeled database. Experiment results found that unsupervised learning presented significant improvements for cross-corpus SER. In Deng et al. (2018), to take advantage of the available unlabeled speech data, they proposed a semi-supervised autoencoder to improve the performance of cross-corpus SER. The proposed method extended a typical unsupervised autoencoder by means of adjoining the supervised learning objective of a deep feed forward network. The extracted acoustic features were the INTERSPEECH-2009 Emotion Challenge feature set. Cross-corpus experiments were implemented by using multiple corpus training via a LOCO scheme. Experimental results showed that the proposed approach obtained promising performance with a very small number of labeled data. In Gideon et al. (2019), the extracted 40 dimensional Mel-filter banks were passed into an adversarial discriminative domain generalization (ADDoG) algorithm to learn more generalized feature representations for cross-corpus SER. Based on the idea of GANs (Goodfellow et al., 2014), ADDoG could make full use of the unlabeled test data to generalize the intermediate feature representation across different datasets. They combined multiple corpora for training and testified the performance of different methods on other corpora via a LOCO scheme. Experiment results showed that ADDoG performed better than CNNs. In Latif et al. (2020), a multi-task semi-supervised adversarial autoencoding (AAE) method was provided for cross-corpus SER. The proposed AAE was a two-step approach. First, semi-supervised learning was implemented in an adversarial autoencoder to generate latent representation. Then, a multi-task learning framework, which considered emotion, speaker and gender identification as auxiliary tasks incorporating with semi-supervised adversarial autoencoding, was built to improve the performance of primary SER task. The spectrograms achieved by a short time Fourier transform (STFT) were employed as inputs of the proposed AAE. They performed cross-corpus experiments with a LOCO scheme on all the used databases. Experiment results demonstrated that the proposed AAE outperformed CNN, CNN+LSTM, as well as DBN.

In recent years, researchers explored ladder network (Valpola, 2015) based semi-supervised methods (Huang et al., 2018; Tao et al., 2019; Parthasarathy and Busso, 2020) for cross-corpus SER and had shown superior results to supervised methods. Here, a ladder network is regarded as an unsupervised DAE trained along with a supervised classification or regression problem. For instance, in Parthasarathy and Busso (2020), a ladder network based semi-supervised method, incorporating with an unsupervised auxiliary task, was presented to reduce the diversity between the source and target domains on cross-corpus SER tasks. The primary task aimed to predict dimensional emotional attributes. The auxiliary task aimed to produce the reconstruction of intermediate feature representations with a DAE. This auxiliary task was trained on a large amount unlabeled data from the target domain in a semi-supervised manner. The ComParE feature set was fed into the ladder network. They conducted cross-corpus experiments with a LOCO scheme. This study indicated that the proposed method achieved superior performance to fully supervised single-task learning (STL) and multi-task learning (MTL) baselines.

In summary, Table 3 presents a summary of the above-mentioned supervised, unsupervised and semi-supervised learning literatures for deep learning methods on cross-corpus SER tasks.

**Table 3:** A brief summary of existing deep cross-corpus SER literatures.

**References**	**Category**	**Input features**	**Methods for cross-corpus**	**Datasets**
Marczewski et al. (2017)	Supervised	54,000 dimensional data points	CNN, LSTM	AFEW, EMO-DB, EMOVO, eNTERFACE05, IEMOCAP
Latif et al. (2018b)	Supervised	eGeMAPS	DBNs	FAU-AIBO, IEMOCAP, EMO-DB, SAVEE, EMOVO
Parry et al. (2019)	Supervised	Mel filterbank coefficients	CNN, LSTM, CNN-LSTM	IEMOCAP, EMOVO, EMO-DB, RAVDESS, SAVEE
Rehman et al. (2020)	Supervised	13 MFCCs	LSTMs, a ramification layer	IEMOCAP, RAVDESS, EMO-DB
Deng et al. (2014b)	Unsupervised	INTERSPEECH-2009	A-DAE	FAU-AIBO, ABC, SUSAS
Deng et al. (2017)	Unsupervised	INTERSPEECH-2009	U-AE	ABC, EMO-DB, SUSAS
Abdelwahab and Busso (2018)	Unsupervised	INTERSPEECH-2013	DANN	IEMOCAP, MSP-IMPROV, MSP-PODCAST
Neumann and Vu (2019)	Unsupervised	128 Mel frequency bands	unsupervised autoencoder and ACNN	IEMOCAP, MSP-IMPROV
Ocquaye et al. (2021)	Unsupervised	spectrogram	three attentive asymmetric CNNs	SAVEE, IEMOCAP, EMO-DB,FAU-AIBO, EMOVO
Chang and Scherer (2017)	Semi-supervised	spectrogram	DCGAN	AMI, IEMOCAP
Deng et al. (2018)	Semi-supervised	INTERSPEECH-2009	Unsupervised autoencoder	FAU-AIBO, ABC, EMO-DB, SUSAS
Gideon et al. (2019)	Semi-supervised	40 dimensional Mel-filter banks	ADDoG	IEMOCAP, MSP-IMPROV
Latif et al. (2020)	Semi-supervised	spectrogram	AAE	IEMOCAP, MSP-IMPROV
Parthasarathy and Busso (2020)	Semi-supervised	INTERSPEECH-2013	ladder network	MSP-PODCAST, IEMOCAP, MSP-IMPROV

## Open Challenges

Although deep learning based cross-corpus SER has made great progress in recent years as mentioned above, there exist still several open challenges that should be addressed in future. In the following, we will discuss these open challenges, and show its potential trends.

One of the most important problems for cross-corpus SER is the generation of natural emotional speech data (Cao et al., 2015). As shown in Table 1, we can see that the majority of emotional databases for cross-corpus SER are acted and recorded in specific silent labs. However, in the real-world sceneries, the collected emotional speech data is usually noisy. In addition, there are also legal and ethical issues when recording the true natural speech emotions. Most existing utterances from natural datasets are collected from talk-shows, call-center recordings, and similar conditions in which the involved participants are informed of the recording. In this case, these natural datasets do not include all emotion categories and may not reflect the true emotions that are felt. Moreover, there is a scarcity for speech emotional datasets in numbers. Considering that deep cross-corpus SER is a data-driven task based on deep learning models with high hyper-parameters, a great number of training data is needed for training sufficiently deep models. Hence, another main challenge for deep cross-corpus SER is the scarcity of enough large emotional datasets.

The second challenge is to integrate more modalities characterized by human emotion expression for cross-corpus emotion recognition (Tzirakis et al., 2021). It is well-known that the typical bimodalities (audio, visual) (Zhang et al., 2017; Zhou et al., 2021), triple modalities (audio, visual, text) (Shoumy et al., 2020), user's physiological responses like electroencephalogram (EEG) and electrocardiogram (ECG) signals (Katsigiannis and Ramzan, 2017; Li et al., 2021), and so on, are highly correlated with human emotion expression. To further improve emotion recognition, it is thus interesting to combine speech clues with other modalities such as visual, text, and physiological clues for multimodal cross-corpus.

Another challenge is the inherent limitation of deep learning techniques. First, although various deep leaning techniques have been successfully employed to capture high-level feature representations for cross-corpus SER, most of deep learning techniques have a large number of network parameters. This makes deep learning techniques usually have very large computation complexity, resulting in its training which demands for large data. To alleviate this problem, it is a promising direction to investigate the application of deep compression and acceleration (Han et al., 2016; Choudhary et al., 2020) techniques such as pruning, trained quantization, and so on, for real-world cross-corpus SER. Additionally, deep learning is a the black-box technique. In particular, due to the used multilayer non-linear architecture, deep learning algorithms are frequently criticized to be non-transparent, and non-explainable. Therefore, it is also a promising research subject to investigate how to understand the explainability and interpretability of deep learning techniques (Fellous et al., 2019; Langer et al., 2021) for cross-corpus SER. In addition, it is also interesting to investigate the performance of recently-developed transformer (Vaswani et al., 2017; Lian et al., 2021) method incorporating with deep learning techniques for cross-corpus SER in our future work.

## Conclusions

This paper has presented an up-to-date and comprehensive review of cross-corpus SER techniques, exhibiting recent advances and perspectives in this area. It has summarized the related speech emotional databases and the applications of deep leaning techniques associated with supervised, unsupervised, semi-supervised learning for cross-corpus SER in recent years. In addition, it highlights several challenging research directions to further improve the performance of cross-corpus SER in future.

## Author Contributions

SZ contributed to the writing and drafted this article. RL and XT contributed to the collection and analysis of existing literatures. XZ contributed to the conception and design of this work and revised this article. All authors contributed to the article and approved the submitted version.

## Funding

This work was supported by Zhejiang Provincial National Science Foundation of China and National Science Foundation of China (NSFC) under Grant Nos. LZ20F020002, LQ21F020002, and 61976149.

## Conflict of Interest

The authors declare that the research was conducted in the absence of any commercial or financial relationships that could be construed as a potential conflict of interest.

## Publisher's Note

All claims expressed in this article are solely those of the authors and do not necessarily represent those of their affiliated organizations, or those of the publisher, the editors and the reviewers. Any product that may be evaluated in this article, or claim that may be made by its manufacturer, is not guaranteed or endorsed by the publisher.

## References

[B1] Abdelwahab M. Busso C. (2018). Domain adversarial for acoustic emotion recognition. IEEE/ACM Trans. Audio Speech Lang. Process. 26, 2423–2435. 10.1109/TASLP.2018.286709927295638

[B2] Akçay M. B. Oguz K. (2020). Speech emotion recognition: emotional models, databases, features, preprocessing methods, supporting modalities, and classifiers. Speech Commun. 116, 56–76. 10.1016/j.specom.2019.12.001

[B3] Alam F. Joty S. Imran M. (2018). Graph based semi-supervised learning with convolution neural networks to classify crisis related tweets, in Twelfth International AAAI Conference on Web and Social Media. (Palo Alto, CA), 556–559.

[B4] Albornoz E. M. Milone D. H. Rufiner H. L. (2011). Spoken emotion recognition using hierarchical classifiers. Comput. Speech Lang. 25, 556–570. 10.1016/j.csl.2010.10.001

[B5] Alloghani M. Al-Jumeily D. Mustafina J. Hussain A. Aljaaf A. J. (2020). A systematic review on supervised and unsupervised machine learning algorithms for data science, in Supervised unsupervised Learn Data Sci. 3–21. 10.1007/978-3-030-22475-2_1

[B6] Anagnostopoulos C.-N. Iliou T. Giannoukos I. (2015). Features and classifiers for emotion recognition from speech: a survey from 2000 to 2011. Artif. Intell. Rev. 43, 155–177. 10.1007/s10462-012-9368-5

[B7] Ballard D. H. (1987). Modular learning in neural networks, in AAAI (Seattle, WA), 279–284.

[B8] Banse R. Scherer K. R. (1996). Acoustic profiles in vocal emotion expression. J. Pers. Soc. Psychol. 70, 614–636. 10.1037/0022-3514.70.3.614 8851745

[B9] Batliner A. Hacker C. Steidl S. Nöth E. D'Arcy S. Russell M. J.. (2004). You Stupid Tin Box”-Children Interacting with the AIBO Robot: A Cross-linguistic Emotional Speech Corpus, in Lrec. 171–174.

[B10] Burkhardt F. Paeschke A. Rolfes M. Sendlmeier W. F. Weiss B. (2005). A database of German emotional speech, in Ninth European Conference on Speech Communication and Technology (Lisbon). 10.21437/Interspeech.2005-446

[B11] Busso C. Bulut M. Lee C.-C. Kazemzadeh A. Mower E. Kim S.. (2008). IEMOCAP: interactive emotional dyadic motion capture database. Lang. Resour. Eval. 42, 335–359. 10.1007/s10579-008-9076-6

[B12] Busso C. Parthasarathy S. Burmania A. AbdelWahab M. Sadoughi N. Provost E. M. (2017). MSP-IMPROV: an acted corpus of dyadic interactions to study emotion perception. IEEE Trans. Affect. Comput. 8, 67–80. 10.1109/TAFFC.2016.251561727295638

[B13] Cao H. Verma R. Nenkova A. (2015). Speaker-sensitive emotion recognition via ranking: studies on acted and spontaneous speech. Comput. Speech Lang. 29, 186–202. 10.1016/j.csl.2014.01.003 25422534 PMC4240517

[B14] Chang J. Scherer S. (2017). Learning representations of emotional speech with deep convolutional generative adversarial networks, in 2017 IEEE International Conference on Acoustics, Speech and Signal Processing (ICASSP) (New Orleans, LA), 2746–2750. 10.1109/ICASSP.2017.7952656

[B15] Chen M. Wei Z. Huang Z. Ding B. Li Y. (2020). Simple and deep graph convolutional networks, in: International Conference on Machine Learning (Long Beach, CA), 1725–1735.

[B16] Chong Y. Ding Y. Yan Q. Pan S. (2020). Graph-based semi-supervised learning: a review. Neurocomputing 408, 216–230. 10.1016/j.neucom.2019.12.130

[B17] Chou H. Lin W. Chang L. Li C. Ma H. Lee C. (2017). NNIME: The NTHU-NTUA Chinese interactive multimodal emotion corpus, in 2017 Seventh International Conference on Affective Computing and Intelligent Interaction (ACII)) (San Antonio, TX), 292–298. 10.1109/ACII.2017.8273615

[B18] Choudhary T. Mishra V. Goswami A. Sarangapani J. (2020). A comprehensive survey on model compression and acceleration. Artif. Intell. Rev. 53, 5113–5155. 10.1007/s10462-020-09816-7

[B19] Costantini G. Iaderola I. Paoloni A. Todisco M. (2014). EMOVO corpus: an Italian emotional speech database, in International Conference on Language Resources and Evaluation (LREC 2014): European Language Resources Association *(ELRA))* 3501–3504.

[B20] Cowie R. Douglas-Cowie E. Savvidou* S. McMahon E. Sawey M. Schröder M. (2000). FEELTRACE': an instrument for recording perceived emotion in real time, in ISCA Tutorial and Research Workshop (ITRW) on Speech and Emotion (Northern Ireland).

[B21] Cowie R. Douglas-Cowie E. Tsapatsoulis N. Votsis G. Kollias S. Fellenz W.. (2001). Emotion recognition in human-computer interaction. IEEE Signal Process. Mag. 18, 32–80. 10.1109/79.91119727295638

[B22] Cunningham P. Cord M. Delany S. J. (2008). Supervised learning, in Machine Learning Techniques for Multimedia eds. Cord M. Cunningham P ( Berlin, Heidelberg: Springer; Cognitive Technologies), p. 21–49. 10.1007/978-3-540-75171-7_2

[B23] Dellaert F. Polzin T. Waibel A. (1996). Recognizing emotion in speech, in: 4th International Conference on Spoken Language Processing (ICSLP'96) (Philadelphia, PA: ISCA), p. 1970–3. 10.1109/ICSLP.1996.608022

[B24] Deng J. Xia R. Zhang Z. Liu Y. Schuller B. (2014a). Introducing shared-hidden-layer autoencoders for transfer learning and their application in acoustic emotion recognition, in 2014 IEEE International Conference on Acoustics, Speech and Signal Processing (ICASSP). p. 4818–22. 10.1109/ICASSP.2014.6854517

[B25] Deng J. Xu X. Zhang Z. Frühholz S. Schuller B. (2017). Universum autoencoder-based domain adaptation for speech emotion recognition. IEEE Signal Process. Lett. 24, 500–504. 10.1109/LSP.2017.267275327295638

[B26] Deng J. Xu X. Zhang Z. Frühholz S. Schuller B. (2018). Semisupervised autoencoders for speech emotion recognition. IEEE/ACM Trans. Audio Speech Lang. Process. 26, 31–43. 10.1109/TASLP.2017.275933827295638

[B27] Deng J. Zhang Z. Eyben F. Schuller B. (2014b). Autoencoder-based unsupervised domain adaptation for speech emotion recognition. IEEE Signal Process. Lett. 21, 1068–1072. 10.1109/LSP.2014.232475927295638

[B28] Dhall A. Ramana Murthy O. V. Goecke R. Joshi J. Gedeon T. (2015). Video and image based emotion recognition challenges in the wild: Emotiw 2015, in Proceedings of the 2015 ACM on international conference on multimodal interaction. (Seattle, WA), p. 423–426.

[B29] Douglas-Cowie E. Cowie R. Sneddon I. Cox C. Lowry O. Mcrorie M.. (2007). The HUMAINE database: Addressing the collection and annotation of naturalistic and induced emotional data, in International Conference on Affective Computing and Intelligent Interaction (Lisbon: Springer), p. 488–500. 10.1007/978-3-540-74889-2_43

[B30] El Ayadi M. Kamel M. S. Karray F. (2011). Survey on speech emotion recognition: features, classification schemes, and databases. Pattern Recognit. 44, 572–587. 10.1016/j.patcog.2010.09.020

[B31] Elman J. L. (1990). Finding structure in time. Cogn. Sci. 14, 179–211. 10.1207/s15516709cog1402_1

[B32] Engberg I. S. Hansen A. V. Andersen O. Dalsgaard P. (1997). Design, recording and verification of a Danish emotional speech database, in Fifth European Conference on Speech Communication and Technology) (Rhodes).

[B33] Eyben F. Scherer K. R. Schuller B. W. Sundberg J. André E. Busso C.. (2016). The Geneva minimalistic acoustic parameter set (GeMAPS) for voice research and affective computing. IEEE Trans. Affect. Comput. 7, 190–202. 10.1109/TAFFC.2015.245741727295638

[B34] Fellous J.-M. Sapiro G. Rossi A. Mayberg H. Ferrante M. (2019). Explainable artificial intelligence for neuroscience: behavioral neurostimulation. Front. Neurosci. 13:1346. 10.3389/fnins.2019.01346 31920509 PMC6923732

[B35] Feng K. Chaspari T. (2020). A review of generalizable transfer learning in automatic emotion recognition. Front. Comput. Sci. 2:9. 10.3389/fcomp.2020.00009

[B36] Feraru S. M. Schuller D. Schuller B. (2015). Cross-language acoustic emotion recognition: an overview and some tendencies, in 2015 International Conference on Affective Computing and Intelligent Interaction (Xi'an: ACII), p. 125–131. 10.1109/ACII.2015.7344561

[B37] Fergus R. Weiss Y. Torralba A. (2009). Semi-Supervised Learning in Gigantic Image Collections, in NIPS (Vancouver, BC: Citeseer), p. 1–9.

[B38] Fragopanagos N. Taylor J. G. (2005). Emotion recognition in human-computer interaction. Neural Netw. 18, 389–405. 10.1016/j.neunet.2005.03.006 15921887

[B39] Garofolo J. S. Lamel L. F. Fisher W. M. Fiscus J. G. Pallett D. S. J. S. (1993). DARPA TIMIT acoustic-phonetic continous speech corpus CD-ROM. NIST Speech Disc. 93:27403. 10.6028/NIST.IR.4930

[B40] Gideon J. McInnis M. Provost E. M. (2019). Improving cross-corpus speech emotion recognition with Adversarial Discriminative Domain Generalization (ADDoG). IEEE Trans. Affect. Comput. 10.1109/TAFFC.2019.2916092. [Epub ahead of print]. 35695825 PMC9173710

[B41] Goodfellow I. Pouget-Abadie J. Mirza M. Xu B. Warde-Farley D. Ozair S.. (2014). Generative adversarial nets, in Advances in Neural Information Processing Systems. (Montreal, QC).

[B42] Gretton A. Smola A. Huang J. Schmittfull M. Borgwardt K. Schölkopf B. (2009). Covariate shift by kernel mean matching. Dataset Shift Mach. Learn. 3, 131–160. 10.7551/mitpress/9780262170055.003.0008

[B43] Grimm M. Kroschel K. Narayanan S. (2008). The Vera am Mittag German audio-visual emotional speech database, in 2008 IEEE International Conference on Multimedia and Expo (Hannover: IEEE), p. 865–868. 10.1109/ICME.2008.4607572

[B44] Han S. Mao H. Dally W. J. (2016). Deep compression: compressing deep neural network with pruning, trained quantization and huffman coding, in International Conference on Learning Representations (ICLR) (Vancouver, BC, Canada).

[B45] Hansen J. H. Bou-Ghazale S. E. (1997). Getting started with SUSAS: A speech under simulated and actual stress database, in Fifth European Conference on Speech Communication and Technology.

[B46] Harati S. Crowell A. Mayberg H. Nemati S. (2018). Depression severity classification from speech emotion, in 2018 40th Annual International Conference of the IEEE Engineering in Medicine and Biology Society (EMBC) (Honolulu, HI: IEEE), p. 5763–5766. 10.1109/EMBC.2018.8513610 30441645

[B47] Hinton G. E. Salakhutdinov R. R. (2006). Reducing the dimensionality of data with neural networks. Science 313, 504–507. 10.1126/science.1127647 16873662

[B48] Hochreiter S. Schmidhuber J. (1997). Long short-term memory. Neural Comput. 9, 1735–1780. 10.1162/neco.1997.9.8.1735 9377276

[B49] Huang J. Li Y. Tao J. Lian Z. Niu M. Yi J. (2018). Speech emotion recognition using semi-supervised learning with ladder networks, in 2018 First Asian Conference on Affective Computing and Intelligent Interaction (ACII Asia) (Beijing), 1–5. 10.1109/ACIIAsia.2018.8470363

[B50] Jackson P. Haq S. (2014). Surrey Audio-Visual Expressed Emotion (savee) Database. Guildford: University of Surrey.

[B51] Katsigiannis S. Ramzan N. (2017). DREAMER: a database for emotion recognition through EEG and ECG signals from wireless low-cost off-the-shelf devices. IEEE J. Biomed. Health Inform. 22, 98–107. 10.1109/JBHI.2017.2688239 28368836

[B52] Kaya H. Karpov A. A. (2018). Efficient and effective strategies for cross-corpus acoustic emotion recognition. Neurocomputing 275, 1028–1034. 10.1016/j.neucom.2017.09.049

[B53] Koolagudi S. G. Reddy R. Yadav J. Rao K. S. (2011). IITKGP-SEHSC: Hindi speech corpus for emotion analysis, in 2011 International Conference on Devices and Communications (ICDeCom) (IEEE). p. 1–5. 10.1109/ICDECOM.2011.5738540

[B54] Kossaifi J. Tzimiropoulos G. Todorovic S. Pantic M. (2017). AFEW-VA database for valence and arousal estimation in-the-wild. Image Vis. Comput. 65, 23–36. 10.1016/j.imavis.2017.02.00131581074

[B55] Kotsiantis S. B. Zaharakis I. Pintelas P. (2007). Supervised machine learning: a review of classification techniques. Emerg. Artif. Intell. Appl. Comput. Eng. 160, 3–24. 10.1007/s10462-007-9052-3

[B56] Krizhevsky A. Sutskever I. Hinton G. E. (2012). Imagenet classification with deep convolutional neural networks, in Advances in Neural Information Processing Systems (Lake Tahoe, NV), 1097–1105.

[B57] Kwon O. Chan K. Hao J. Lee T. (2003). Emotion recognition by speech signals, in EUROSPEECH-2003 (ISCA). p. 125–128.

[B58] Langer M. Oster D. Speith T. Hermanns H. Kästner L. Schmidt E.. (2021). What do we want from Explainable Artificial Intelligence (XAI)?–A stakeholder perspective on XAI and a conceptual model guiding interdisciplinary XAI research. Artif. Intell. 296, 103473. 10.1016/j.artint.2021.103473

[B59] Latif S. Qayyum A. Usman M. Qadir J. (2018a). Cross lingual speech emotion recognition: Urdu vs. western languages, in 2018 International Conference on Frontiers of Information Technology (FIT) (IEEE). p. 88–93. 10.1109/FIT.2018.00023

[B60] Latif S. Rana R. Khalifa S. Jurdak R. Epps J. Schuller B. W. (2020). Multi-Task semi-supervised adversarial autoencoding for speech emotion recognition. IEEE Trans. Affect. Comput. 1–1. 10.1109/TAFFC.2020.298366927295638

[B61] Latif S. Rana R. Younis S. Qadir J. Epps J. (2018b). Transfer learning for improving speech emotion classification accuracy. arXiv preprint arXiv 1801.06353. 10.21437/Interspeech.2018-1625

[B62] LeCun Y. Bengio Y. Hinton G. (2015). Deep learning. Nature 521, 436–444. 10.1038/nature14539 26017442

[B63] Lee D. D. Seung H. S. (1999). Learning the parts of objects by non-negative matrix factorization. Nature 401, 788–791. 10.1038/44565 10548103

[B64] Li W. Huan W. Hou B. Tian Y. Zhang Z. Song A. (2021). Can emotion be transferred?–A review on transfer learning for EEG-Based Emotion Recognition. IEEE Trans. Cogn. Dev. Syst. 10.1109/TCDS.2021.309884233014031

[B65] Li W. Zhang Y. Fu Y. (2007). Speech emotion recognition in e-learning system based on affective computing, in Third International Conference on Natural Computation (ICNC-2007) (Haikou: IEEE), 809–813. 10.1109/ICNC.2007.677

[B66] Li Y. Tao J. Chao L. Bao W. Liu Y. (2017). CHEAVD: a Chinese natural emotional audio–visual database. J. Ambient Intell. Humaniz. Comput. 8, 913–924. 10.1007/s12652-016-0406-z

[B67] Lian Z. Liu B. Tao J. (2021). CTNet: Conversational transformer network for emotion recognition. IEEE/ACM Trans. Audio Speech Lang. Process. 29, 985–1000. 10.1109/TASLP.2021.304989827295638

[B68] Ligthart A. Catal C. Tekinerdogan B. (2021). Analyzing the effectiveness of semi-supervised learning approaches for opinion spam classification. Appl. Soft Comput. 101:107023. 10.1016/j.asoc.2020.107023

[B69] Likas A. Vlassis N. Verbeek J. J. (2003). The global k-means clustering algorithm. Pattern Recognit. 36, 451–461. 10.1016/S0031-3203(02)00060-2

[B70] Lin Y. Gau S. S. Lee C. (2020). A multimodal interlocutor-modulated attentional BLSTM for classifying autism subgroups during clinical interviews. IEEE J. Sel. Top. Signal Process. 14, 299–311. 10.1109/JSTSP.2020.297057827295638

[B71] Liu N. Zhang B. Liu B. Shi J. Yang L. Li Z.. (2021). Transfer subspace learning for unsupervised cross-corpus speech emotion recognition. IEEE Access 9, 95925–95937. 10.1109/ACCESS.2021.309435527295638

[B72] Liu N. Zong Y. Zhang B. Liu L. Chen J. Zhao G.. (2018). Unsupervised Cross-Corpus Speech Emotion Recognition Using Domain-Adaptive Subspace Learning, in 2018 IEEE International Conference on Acoustics, Speech and Signal Processing (ICASSP) 5144–5148. 10.1109/ICASSP.2018.846184827295638

[B73] Livingstone S. R. Russo F. A. (2018). The Ryerson Audio-Visual Database of Emotional Speech and Song (RAVDESS): a dynamic, multimodal set of facial and vocal expressions in North American English. PLoS ONE 13:e0196391. 10.1371/journal.pone.0196391 29768426 PMC5955500

[B74] Lotfian R. Busso C. (2019). Building naturalistic emotionally balanced speech corpus by retrieving emotional speech from existing podcast recordings. IEEE Trans. Affect. Comput. 10, 471–483. 10.1109/TAFFC.2017.273699927295638

[B75] Luengo I. Navas E. Hernáez I. (2010). Feature analysis and evaluation for automatic emotion identification in speech. IEEE Trans. Multimedia 12, 490–501. 10.1109/TMM.2010.205187227295638

[B76] Luo H. Han J. (2019). Cross-corpus speech emotion recognition using semi-supervised transfer non-negative matrix factorization with adaptation regularization, in INTERSPEECH. 3247–3251. 10.21437/Interspeech.2019-2041

[B77] Luo H. Han J. (2020). Nonnegative matrix factorization based transfer subspace learning for cross-corpus speech emotion recognition. IEEE/ACM Trans. Audio Speech Lang. Process. 28, 2047–2060. 10.1109/TASLP.2020.300633127295638

[B78] Mao Q. Xu G. Xue W. Gou J. Zhan Y. (2017). Learning emotion-discriminative and domain-invariant features for domain adaptation in speech emotion recognition. Speech Commun. 93, 1–10. 10.1016/j.specom.2017.06.006

[B79] Marczewski A. Veloso A. Ziviani N. (2017). Learning transferable features for speech emotion recognition, in Proceedings of the on Thematic Workshops of ACM Multimedia (Mountain View, CA), 529–536. 10.1145/3126686.3126735

[B80] Martin O. Kotsia I. Macq B. Pitas I. (2006). The eNTERFACE'05 audio-visual emotion database, in 22nd International Conference on Data Engineering Workshops (ICDEW'06) (IEEE). p. 8-8. 10.1109/ICDEW.2006.145

[B81] Morrison D. Wang R. De Silva L. C. (2007). Ensemble methods for spoken emotion recognition in call-centres. Speech Commun. 49, 98–112. 10.1016/j.specom.2006.11.004

[B82] Neumann M. Vu N. T. (2019). Improving Speech Emotion Recognition with Unsupervised Representation Learning on Unlabeled Speech, in ICASSP 2019 - 2019 IEEE International Conference on Acoustics, Speech and Signal Processing (ICASSP) (Brighton), 7390–7394. 10.1109/ICASSP.2019.8682541

[B83] Nicholson J. Takahashi K. Nakatsu R. (2000). Emotion recognition in speech using neural networks. Neural Computing Appl. 9, 290–296. 10.1007/s005210070006

[B84] Nwe T. L. Foo S. W. De Silva L. C. (2003). Speech emotion recognition using hidden Markov models. Speech Commun. 41, 603–623. 10.1016/S0167-6393(03)00099-2

[B85] Ocquaye E. N. Mao Q. Xue Y. Song H. (2021). Cross lingual speech emotion recognition via triple attentive asymmetric convolutional neural network. Int. J. Intelligent Syst. 36, 53–71. 10.1002/int.2229125855820

[B86] Otter D. W. Medina J. R. Kalita J. K. (2020). A survey of the usages of deep learning for natural language processing. IEEE Transact. Neural Netw. Learn. Syst. 32, 604–624. 10.1109/TNNLS.2020.2979670 32324570

[B87] Parry J. Palaz D. Clarke G. Lecomte P. Mead R. Berger M.. (2019). Analysis of deep learning architectures for cross-corpus speech emotion recognition, in Interspeech-2019 (Graz), 1656–1660. 10.21437/Interspeech.2019-2753

[B88] Parthasarathy S. Busso C. (2020). Semi-supervised speech emotion recognition with ladder networks. IEEE/ACM Transact. Audio Speech Language Proc. 28, 2697–2709. 10.1109/TASLP.2020.302363227295638

[B89] Picard R. W. (2010). Affective computing: from laughter to IEEE. IEEE Transact. Affect. Computing 1, 11–17. 10.1109/T-AFFC.2010.1027295638

[B90] Purwins H. Li B. Virtanen T. Schlüter J. Chang S.-Y. Sainath T. (2019). Deep learning for audio signal processing. IEEE J. Sel. Top. Signal Process. 13, 206–219. 10.1109/JSTSP.2019.290870027295638

[B91] Ramakrishnan S. El Emary I. M. (2013). Speech emotion recognition approaches in human computer interaction. Telecommun. Syst. 52, 1467–1478. 10.1007/s11235-011-9624-z

[B92] Rehman A. Liu Z. T. Li D. Y. Wu B. H. (2020). Cross-corpus speech emotion recognition based on hybrid neural networks, in 2020 39th Chinese Control Conference (CCC) (Shenyang), 7464–7468. 10.23919/CCC50068.2020.9189368

[B93] Samani H. A. Saadatian E. (2012). A multidisciplinary artificial intelligence model of an affective robot. Int. J. Advanced Robotic Syst. 9, 1–11. 10.5772/45662

[B94] Saxena A. Prasad M. Gupta A. Bharill N. Patel O. P. Tiwari A.. (2017). A review of clustering techniques and developments. Neurocomputing 267, 664–681. 10.1016/j.neucom.2017.06.053

[B95] Schmidhuber J. (2015). Deep learning in neural networks: an overview. Neural Netw. 61, 85–117. 10.1016/j.neunet.2014.09.003 25462637

[B96] Schuller B. Arsic D. Rigoll G. Wimmer M. Radig B. (2007). Audiovisual behavior modeling by combined feature spaces, in 2007 IEEE International Conference on Acoustics, Speech and Signal Processing-ICASSP'07 (Honolulu, HI: IEEE), p. II-733-II-736. 10.1109/ICASSP.2007.366340

[B97] Schuller B. Müller R. Eyben F. Gast J. Hörnler B. Wöllmer M.. (2009a). Being bored? Recognising natural interest by extensive audiovisual integration for real-life application. Image Vision Computing 27, 1760–1774. 10.1016/j.imavis.2009.02.013

[B98] Schuller B. Steidl S. Batliner A. (2009b). The interspeech 2009 emotion challenge, in Tenth Annual Conference of the International Speech Communication Association (Brighton). 10.21437/Interspeech.2009-103

[B99] Schuller B. Steidl S. Batliner A. Burkhardt F. Devillers L. Müller C.. (2010a). The INTERSPEECH 2010 paralinguistic challenge, in Eleventh Annual Conference of the International Speech Communication Association (Makuhari). 10.21437/Interspeech.2010-739

[B100] Schuller B. Steidl S. Batliner A. Vinciarelli A. Scherer K. Ringeval F.. (2013). The INTERSPEECH 2013 computational paralinguistics challenge: Social signals, conflict, emotion, autism, in Proceedings INTERSPEECH 2013, 14th Annual Conference of the International Speech Communication Association (Lyon). 10.21437/Interspeech.2013-56

[B101] Schuller B. Vlasenko B. Eyben F. Wöllmer M. Stuhlsatz A. Wendemuth A.. (2010b). Cross-corpus acoustic emotion recognition: Variances and strategies. IEEE Transact. Affect. Computing 1, 119–131. 10.1109/T-AFFC.2010.827295638

[B102] Schuller B. W. (2018). Speech emotion recognition: two decades in a nutshell, benchmarks, and ongoing trends. Commun. ACM 61, 90–99. 10.1145/3129340

[B103] Sen P. C. Hajra M. Ghosh M. (2020). Supervised classification algorithms in machine learning: A survey and review, in Emerging Technology in Modelling and Graphics (Springer). p. 99–111. 10.1007/978-981-13-7403-6_11

[B104] Shoumy N. J. Ang L.-M. Seng K. P. Rahaman D. M. Zia T. (2020). Multimodal big data affective analytics: a comprehensive survey using text, audio, visual and physiological signals. J. Netw. Computer Applicat. 149:102447. 10.1016/j.jnca.2019.102447

[B105] Song P. Zhang X. Ou S. Liu J. Yu Y. Zheng W. (2016a). Cross-corpus speech emotion recognition using transfer semi-supervised discriminant analysis, in 2016 10th International Symposium on Chinese Spoken Language Processing (ISCSLP)), 1–5. 10.1109/ISCSLP.2016.791839527295638

[B106] Song P. Zheng W. Ou S. Zhang X. Jin Y. Liu J.. (2016b). Cross-corpus speech emotion recognition based on transfer non-negative matrix factorization. Speech Commun. 83, 34–41. 10.1016/j.specom.2016.07.010

[B107] Staroniewicz P. Majewski W. (2009). Polish emotional speech database–recording and preliminary validation, in Cross-Modal Analysis of Speech, Gestures, Gaze and Facial Expressions (Springer). p. 42–49. 10.1007/978-3-642-03320-9_5

[B108] Steininger S. Schiel F. Dioubina O. Raubold S. (2002). Development of user-state conventions for the multimodal corpus in smartkom, in Proc. Workshop on Multimodal Resources and Multimodal Systems Evaluation (Las Palmas), 33–37.

[B109] Tao J.-H. Huang J. Li Y. Lian Z. Niu M.-Y. (2019). Semi-supervised ladder networks for speech emotion recognition. Int. J. Automation Comput. 16, 437–448. 10.1007/s11633-019-1175-x

[B110] Tzirakis P. Chen J. Zafeiriou S. Schuller B. (2021). End-to-end multimodal affect recognition in real-world environments. Information Fusion 68, 46–53. 10.1016/j.inffus.2020.10.011

[B111] Usama M. Qadir J. Raza A. Arif H. Yau K.-L. A. Elkhatib Y.. (2019). Unsupervised machine learning for networking: Techniques, applications and research challenges. IEEE Access 7, 65579–65615. 10.1109/ACCESS.2019.291664827295638

[B112] Valpola H. (2015). From neural PCA to deep unsupervised learning, in Advances in Independent Component Analysis and Learning Machines, eds Bingham E. Kaski S. Laaksonen J. Lampinen J. (Academic Press). p. 143–171. 10.1016/B978-0-12-802806-3.00008-7

[B113] Van Der Maaten L. Postma E. Van den Herik J. (2009). Dimensionality reduction: a comparative. J. Mach. Learn. Res. 10, 66–71.

[B114] van Engelen J. E. Hoos H. H. (2020). A survey on semi-supervised learning. Mach. Learn. 109, 373–440. 10.1007/s10994-019-05855-6

[B115] Vaswani A. Shazeer N. Parmar N. Uszkoreit J. Jones L. Gomez A. N.. (2017). Attention is all you need, in Advances in Neural Information Processing Systems (Long Beach, CA), 5998–6008.

[B116] Ververidis D. Kotropoulos C. (2005). Emotional speech classification using Gaussian mixture models, in IEEE International Conference on Multimedia and Expo (ICME'05) (Amsterdam), 2871–2874. 10.1109/ISCAS.2005.1465226

[B117] Wold S. Esbensen K. Geladi P. (1987). Principal component analysis. Chemometr. Intelligent Lab. Syst. 2, 37–52. 10.1016/0169-7439(87)80084-9

[B118] Wöllmer M. Eyben F. Reiter S. Schuller B. Cox C. Douglas-Cowie E.. (2008). Abandoning emotion classes-towards continuous emotion recognition with modelling of long-range dependencies, in Proc. 9th Interspeech 2008 Incorp. 12th Australasian Int. Conf. on Speech Science and Technology SST 2008 (Brisbane), 597–600. 10.21437/Interspeech.2008-192

[B119] Wu T. Yang Y. Wu Z. Li D. (2006). Masc: a speech corpus in mandarin for emotion analysis and affective speaker recognition, in 2006 IEEE Odyssey-the Speaker and Language Recognition Workshop (San Juan: IEEE), p. 1–5. 10.1109/ODYSSEY.2006.248084

[B120] Wu X. Sahoo D. Hoi S. C. (2020). Recent advances in deep learning for object detection. Neurocomputing 396, 39–64. 10.1016/j.neucom.2020.01.085

[B121] Yildirim S. Narayanan S. Potamianos A. (2011). Detecting emotional state of a child in a conversational computer game. Comput. Speech Lang. 25, 29–44. 10.1016/j.csl.2009.12.004

[B122] Zhalehpour S. Onder O. Akhtar Z. Erdem C. E. (2016). BAUM-1: a spontaneous audio-visual face database of affective and mental states. IEEE Transact. Affect. Comput. 8, 300–313. 10.1109/TAFFC.2016.255303827295638

[B123] Zhang J. T. Jia H. (2008). Design of speech corpus for mandarin text to speech, in The Blizzard Challenge 2008 Workshop (Brisbane, QLD).

[B124] Zhang S. Zhang S. Huang T. Gao W. Tian Q. (2017). Learning affective features with a hybrid deep model for audio–visual emotion recognition. IEEE Transact. Circuits Syst. Video Tech. 28, 3030–3043. 10.1109/TCSVT.2017.271904327295638

[B125] Zhang S. Zhao X. (2013). Dimensionality reduction-based spoken emotion recognition. Multimed. Tools Appl. 63, 615–646. 10.1007/s11042-011-0887-x

[B126] Zhang S. Zhao X. Lei B. (2013). Speech emotion recognition using an enhanced kernel isomap for human-robot interaction. Int. J. Adv. Robotic Syst. 10, 1–7. 10.5772/55403

[B127] Zhang Z. Weninger F. Wöllmer M. Schuller B. (2011). Unsupervised learning in cross-corpus acoustic emotion recognition, in 2011 IEEE Workshop on Automatic Speech Recognition and Understanding (Waikoloa, HI), 523–528. 10.1109/ASRU.2011.6163986

[B128] Zhao X. Zhang S. (2015). Spoken emotion recognition via locality-constrained kernel sparse representation. Neural Comput. Appl. 26, 735–744. 10.1007/s00521-014-1755-1

[B129] Zhou H. Du J. Zhang Y. Wang Q. Liu Q. F. Lee C. H. (2021). Information fusion in attention networks using adaptive and multi-level factorized bilinear pooling for audio-visual emotion recognition. IEEE/ACM Transact. Audio Speech Language Processing 29, 2617–2629. 10.1109/TASLP.2021.309603727295638

